# Abortion with Retained Placenta*Read before the Baltimore Medical and Surgical Society.

**Published:** 1881-12

**Authors:** Wilmer Brinton

**Affiliations:** Baltimore


### Independent Practitioner

VOL. II.——*December, 1881.*——No. 12.

ARTICLE I.

### ABORTION WITH RETAINED PLACENTA.*

BY WILMER BRINTON, M. D., BALTIMORE.

When we come to consider how often in practice we are called upon to attend patients who are aborting, or if you please to call it, miscarrying, and how often the medical man looks upon the treatment of all these cases as a mere matter of routine, requiring no consideration in special cases; and having recently several cases coming under my care, after having been attended by other medical men during the abortion, who had been left by their attendant for well—yet really left on the brink of a precipice, only a step as it were, if not between life and death—between health and years of future ill-health; when we remember that it is fashionable and popular at the present time to have law suits for damages from which even medical men are not exempt, the question might come up in the future, how far a medical man might be legally responsible, and what amount of damage be awarded in a case of hemorrhage with loss of life, caused by a retained placenta, where the medical man in attendance had dismissed his patient a day, week or month previous, with the assurance that every thing was "all right."

These and a great many other facts have induced me to bring to your notice to-night the well written and often discussed subject of "abortion with retained placenta;" not expecting to bring any facts especially new for your consideration, but hoping the relation of a few

* Read before the Baltimore Medical and Surgical Society.

cases which I propose to do later on, will excite a discussion that will prove beneficial to us all. Abortion, as understood in this paper, meaning the premature separation and expulsion of the contents of the pregnant uterus as an accident of pregnancy, is far from uncommon, although its relative frequency, as compared with that of completed gestation, has been very differently estimated by accoucheurs. Of the importance and frequency of abortion, Playfair, in his last edition, says: "The premature expulsion of the fœtus is an event of great frequency. The number of fœtal lives thus lost is enormous. There are few multiparas who have not aborted at one time or another of their lives." Hegar estimates that about one abortion occurs to every 8 or 10 deliveries at full term. Whitehead has calculated that at least 90 per cent. of married women who lived to the change of life had aborted. Abortion is more liable to occur in the earlier than in the later months of pregnancy, and it would also appear to occur more readily at the periods corresponding to those of the menstrual discharge. It may be induced by numerous causes, both of a local and general nature, malformation of the pelvis, accidental injuries, and the disease and displacements to which the uterus is liable on the one hand, and on the other, various morbid conditions of the ovum or placenta leading to the death of the fœtus, are among the direct local causes of abortion. The general causes embrace certain states of the system which are apt to exercise a more or less direct influence upon the progress of utero-gestation. A deteriorated condition of health, whether hereditary or as the results of habits of life, certainly predisposes to the occurrence of the accident under consideration. Syphilis is known to be a frequent cause of the death of the fœtus. Many diseases arising in the course of pregnancy, act as direct exciting causes of abortion; more particularly the eruptive fevers and acute inflammatory affections. Prolonged irritation in other organs may, by reflex action, excite the uterus to expel its contents; strong impressions made upon the nervous system, as sudden shocks and mental emotions occasionally have a similar effect.

Dr. Theophilus Parvin, in the *American Practitioner*, for July, 1881, thinks coition during pregnancy is the cause of one half of the cases of spontaneous abortion, and opposes the same during pregnancy on the ground that it is against the law of nature, as indicated among the lower animals, that it is generally odious and often painful to the woman, and that in many instances it probably increases the leucorrhœa, nausea and vomiting of the early months.

Another supposed factor in producing abortion is the use of certain medicinal substances, notably among them, savine, tansy, and especially ergot; but a great many cases have come under my notice where these remedies have been used in large quantities with negative results.

There is no doubt that sometimes we have abortion produced by strong purgative medicines, which have, of course, no direct action on the uterus; but which act, indirectly, by irritating the alimentary canal. Many cases of abortion occur without apparent cause, but in such instances the probability is that some morbid condition of the interior of the uterus exists, and the same may be said of many of those cases where the disposition to abortion has become habitual. The tendency, however, to the recurrence of abortion in persons who have previously miscarried is well known, and should ever be borne in mind with the view of avoiding any causes likely to lead to a repetition of the accident.

The last cause which I shall mention is *criminal abortion;* for unfortunately as medical practitioners, we are often brought face to face with the fact that woman (not of the lower class alone) will permit, and a medical man perform the horrible deed—*the murder of a helpless being;* by doing so, bringing disgrace on the fair name of womanhood and dishonor to the medical profession. As I understand the laws of Maryland on this point, the woman on whom the abortion has been produced makes herself a party to the crime, otherwise exposure and punishment of the wretches who commit such acts would be far more frequent and certain.

SYMPTOMS OF ABORTION.—Among so many practical men, it would seem hardly necessary to speak of the symptoms of abortion, but as it never does harm to reiterate important facts, I would say that in a patient threatened with a miscarriage, we have, as a rule, the following train of symptoms, subject to some variation in each individual case. The patient complains of aching or pain in the back, accompanied with a sense of languor, uneasiness and weariness; after these preliminary symptoms have lasted for some time, those of labor supervene, and in most cases they do not differ much from those of labor at full term; indeed, I have had patients who suffer more from their pain when aborting than they do at full term. A slight discharge of blood and mucus from the vagina is observed. a digital examination will now reveal the condition of the os, and if the abortion is inevitable we will find it patulous, and either the placenta or fœtus presenting.

The stomach frequently becomes irritable and discharges its contents, the pulse is quickened, the skin hot, voluntary efforts are made in aid of the uterus, and ultimately the contents of the womb, or a portion of them, are expelled. Although all these symptoms are present, yet they are as specified above, subject to variation in some cases; at times we have alarming hemorrhage, even before there is much pain; then we have the ovum passed in some cases without either much pain or hemorrhage, followed by a speedy recovery; we see this chiefly in persons who have acquired the habit of abortion.

Results of Abortion.—It must be a well known fact to all medical men and especially to gynæcologists, that abortion is frequently the cause of chronic uterine diseases of various kinds, and enlarged or displaced uterus may be dated from a certain miscarriage. This fact should lead us all to pay much attention to our patients during and after abortion. In my own individual practice I try to impress upon my patients the importance of taking fully as much rest in bed as they do at labor at full term; yet among the lower classes this is a hard matter to do, and it is quite a frequent occurrence for me to see a woman aborting to-day, and to-morrow washing; looking upon the occurrence as a matter of no consequence whatever; yet, the day is probably coming when they will have to come into the tender hands of some specialist, with some form of uterine disease, the result of neglect at the time of aborting. But as this paper is announced to refer more especially to "Abortion with Retained Placenta," I will refer to the results following retained placenta. There is no doubt in my mind, that often in abortion, when the patient is not more than six to eight weeks advanced in pregnancy, we often have the retained placenta or portion of retained placenta dissolved and discharged with the lochia, without causing any special trouble. All of us can call to mind patients consulting us about a discharge lasting two, three, five or six weeks after the occurrence. These are, no doubt, cases to which we have referred; but this favorable termination is not always the case, and I think it is a good axiom for every medical man to keep in mind in these cases, *that as long as we have a portion of placenta retained our patient is in danger.* In danger from what? From hemorrhage more especially, or it may be *septicæmia, metritis or perimetritis*, or kindred trouble setting in; sometimes we have the placenta or portions of it which are retained in the uterus, forming organic connection with the walls of that organ, causing more or less hemorrhage with its train of symptoms for months or years, until some of our gynæcological brethren come along who call it a polypus or something else, and charge rightfully a large fee for removing it.

How often in practice do we see patients with a profound anæmic condition, dating their bad health from the time of their last miscarriage, with the history of a continued loss of blood for one week, one month, sometimes three months or longer, yet the medical man in attendance giving drugs, and assuring them everything will be all right, yet never examining his patient the proper way, and finding out and removing the true cause of the mischief, until his patient becoming dissatisfied and disgusted, either secretly or openly, leaves her family physician and consults the specialist or some other practitioner of medicine, and the cause of all her trouble found and removed; but unfortunately in some cases, not until almost irreparable damage has been done, for admitting as a rule the wonderful recuperative power of woman after the loss of large quantities of blood, yet at times it must be a prominent factor in causing not only present trouble, but future ill-health, especially when our patient is predisposed to phthisis, scrofulous or cancerous diseases.

With your permission, I propose to speak of five cases bearing on the subject under consideration, which have come under my notice within the last six months, with the exception of case 1st. These cases being interesting and instructive to me from the fact that with the exception of case 1st, they had been in the hands of medical gentlemen who had dismissed them, assuring them that "they were all right." Other cases have also come under my notice during my six years' practice of physic; but not having kept a record of them, I will only speak of those occurring more recently.

Case 1st. June, 1877, Mrs. A., age 28, mother of two children, supposed herself to be near three months pregnant in June, 1877; one evening while visiting a store in vicinity of her residence, in some manner fell into an opening in the street which had been carelessly left open by some employees of the city, who were repairing and repaving the streets; her cries brought some assistance, and she was helped home; during the night she had some discharge of blood and mucus with severe pains at times which grew worse as the morning came. Dr. F. O. Donnally, now deceased, then living opposite my residence, was summoned to see her. By the time Dr. Donnally reached his patient she had aborted. The fœtus was awaiting his arrival, also the larger portion of the placenta, (all of it the doctor thought at the time) and so informed his patient that she would soon be all right; but from this time until August, a period of almost two months, his patient kept losing large amounts of blood; one day she was about, the next in bed, the doctor still being busy engaged in giving her drugs, until, as he informed me afterwards, he had given ergot and every astringent in the materia medica a fair trial.

My acquaintance with the case commenced on an excessively hot night in August, 1877, when I received a hasty summons from the doctor asking my assistance in the case. Upon my arrival there a little after midnight, I found the woman had been having a severe hemorrhage during the afternoon and evening, which was continuing to some extent in spite of all the doctor's efforts; her pulse was 150, temp. 103½ cold sweats over her body, breathing rapid, pain over the uterus and vicinity, in fact, her general condition was alarming. Upon consultation we determined to explore the cavity ot the uterus; chloroform was administered, and she was kept under its influence for over forty minutes. Upon making a digital examination I found the os dilated and dilatable, and after some slight efforts I succeeded in introducing two fingers into the cavity of the uterus, where I detected a foreign body attached to the anterior wall of the uterus, midway between the fundus and internal os, and as my fingers were considerable cramped for the want of room, it was with exertion upon my part that I was enabled to detach the mass in two pieces, one the size of a walnut, the other about the size of a small peanut. After the removal of the mass I used my finger nails as a curette, and scraped the uterine walls at the place of adhesion. The vagina and uterus were then syringed out with a weak solution of carbolic acid; at once the hemorrhage ceased; the patient rallied from the effects of chloroform nicely. I only saw this patient for two or three days following the removal of the retained and adherent placental mass, but her physician, Dr. Donnally, informed me that she had considerable metritis for some ten days, but under the continued use of carbolic acid injections and opium and quinia internally, she made a good recovery without any more hemorrhages.

Case 2d. April, 1881, was called to see Mrs. B., a woman of the age of 40 years, of rather a fine physique, although somewhat anæmic in appearance, had several children, the youngest living being a young woman age 17 years. My patient informed me that she had been regular until about fourteen months previous, when "she lost her periods" for a month, and when they came on her again at the end of the second month she had considerable hemorrhage, and passed some clots of blood. From that time until I was called to see her, a period of nearly twelve months, she had been losing a great amount of blood, not only during her regular menstrual period, but often in the interval. All this time she was still going around and attending to her household duties. Her family physician was sent for several times, during the twelve months, but, as my patient said, always made light of it, saying it was the "change of life working on her," prescribed for her, and would not return again unless sent for. To me, while she presented as specified before a somewhat anæmic appearance, I could hardly credit her account of the immense loss of blood which she had at times lost. She also complained of headache and intense pain in the back. Upon a digital examination I found the os dilated and dilatable, but not enough to introduce my finger any distance without causing some pain, so for the time desisted. Prescribed ergot gtts. xxx. and kali bromidi grs. xv. four times a day, and absolute rest in bed until I should see her again within the next two or three days. Just at this time the loss of blood was slight, but she informed me that just a few days previous she had an "immense" hemorrhage, that being the immediate cause of my being sent for. Two days after my seeing her I was sent for in a great hurry to see Mrs. B. again, but not being at my office at the time, it was over an hour after the message was received before I arrived at my patient's house, and found her almost in a collapsed condition, pulse hardly perceptible at the wrist, lips blue, extremities cold, and a cold sweat in large drops all over the body, in fact I found my patient as I thought in an alarming condition, the cause of all this being a hemorrhage of the most serious character, the evidence of the same being on the floor, having been in such quantities as to pass through the bedding. I found the bleeding going on to some extent, which I soon controlled by ice in the vagina, and ergot and brandy by the mouth and hypodermically, after which I tamponed the vagina with Monsel's sol. of iron diluted with water, on cotton. Two days afterwards, there being no hemorrhage in the interval, I removed this tampon and made another digital examination, and found the os much more dilated than on a previous occasion. With this examination I detected a foreign body in the cavity of the uterus, but could not get a hold of it with my fingers. I introduced Howard's bivalve speculum, and with a long pair of placental forceps I removed a putrid and offensive mass about the size of a small lemon in the aggregate. Again introducing my finger into the uterus and satisfying myself all had been removed, syringed out the the uterine cavity and vagina with a weak solution of carbolic acid, tamponed the vagina again, gave my patient grs. x. of quinia and I. gr· of opium, and ordered absolute rest for a few days. In a little over a week she was attending to her usual duties, minus some of the heavier work, has been regular ever since, and with the exception of a slight malarial attack in August, has had no need of medical attention since. No hemorrhage took place after the removal of the foreign body, and from that time the intense pain complained of in the back, also disappeared to a great extent. My diagnosis in the case was *a retained placenta* from an abortion which took place over a year before.

CASE 3d. Was called to see Mrs. C., September, 1881, her history being about as follows: Age 18, married four months. Two weeks previous to my being sent for, while washing, was taken unwell (she considering herself at this time to be about three months pregnant) large clots passed, and she went to her bed, when she had quite an extensive hemorrhage. Some hours after this a medical man of questionable repute was called in to see her, examined her, called again the next day, told her she was all right and could get up and go to work. She felt comparatively well for two or three days subsequent to this, and did resume, to some extent, her domestic duties, then had a considerable loss of blood, accompanied with some pain, which continuing, I was sent for. Upon making a digital examination found the os dilatable, some tenderness in the left iliac region, pulse 100, temp. 101½, with a discharge of an offensive nature from the vagina. I prescribed for her opium, grs. ½ and quinia, grs. iij. every three hours, and absolute rest in bed. The next day examined with bivalve speculum, and with my fingers and long forceps removed from the uterus without much trouble several pieces of retained placenta, amounting in the aggregate to a large walnut. The uterine cavity and vagina were syringed out with a weak solution of carbolic acid for the next three or four days, and the opium and quinia continued in smaller doses. Within a week I discharged my patient enjoining upon her to take all the rest possible until after her next menstrual period, and left her a tonic consisting of iron and quinia. Was again called to see her about one month afterwards, found her menses upon her, accompanied with pain, especially in the left iliac region; opium and rest were again ordered, and within a day or two all these unpleasant symptoms passed away; from then until the present time she has had no further trouble.

CASE 4TH. Was called out of bed one morning in June, 1881, to see Mrs. D., found my patient to be a large and fine-looking Irish lady, with an alarming hemorrhage from the uterus, which I soon controlled with ergot and ice in the vagina; she informed me that she was about 30 years of age, the mother of several children, all dead but one; had a miscarriage about fourteen days previous, being then nearly four months advanced in pregnancy: the abortion took place before her family physician arrived, although he had been sent for early, but was delayed in his arrival. She was kept in bed for a week, then her physician who, by the way, is a well-known one, assured her that every thing was all right, advised her to resume her usual duties, although Mrs. McN. assured me that she kept insisting to the doctor that the after-birth had never came away, but she said he laughed at her, and wanted to know "who knew best, him or her." So in this manner she was silenced, and, as mentioned before, resumed her usual duties, feeling no bad effects whatever until the morning when she had the hemorrhage, which came on her without any exertion on her part, or without any premonition of its appearance. After the hemorrhage was under control, upon an examination, I could feel the uterus through the abdominal walls, being very much larger than it ought to be in the non-pregnant state, feeling very much like a womb does after delivery at full term. Absolute rest and ergot were ordered for my patient; two days afterwards was again called out of bed to see her, and found another extensive hemorrhage going on, which I soon controlled as before, with ergot hypodermically and by the mouth, and ice in the vagina. Before leaving my patient I tamponed the vagina with cotton. On the following day removed the same, and found there had been considerable bleeding; introduced a bivalve speculum, and with a short pair of forceps and fingers I made my diagnosis of retained placenta, and removed several small pieces, but she complaining of pain and exhaustion, I desisted from my attempt for the day. Recognizing that I would need some assistance in the case, I called on my friend, Dr. J. H. Scarff, who responded early next morning, and on the fourth day after the first hemorrhage she was placed in Sims' position, chloroformed, and Dr. Scarff and myself alternated in removing the placenta, it being done by introducing the hand into the vagina and finger or fingers into the cavity of the uterus, and removing the placenta (which we found firmly attached to the walls of the uterus) in pieces. Judging from the amount of placenta removed, I would suppose the woman was right in believing herself to be fully four months pregnant when the abortion took place, and why this large placenta escaped the notice of her medical attendant is beyond my comprehension. After the removal of the placenta the parts were syringed with a weak solution of carbolic acid and the vagina tamponed, but from this time there was no more hemorrhage, but within thirty-six hours a severe chill occurred, followed with high fever, the temperature being 105½, pulse 150, and my patient had a severe attack of metritis and cellulitis, and she was alarmingly ill for two weeks, but thanks to about 800 grs. of quinia and nearly a drachm of opium, taken during the time of her illness, she made a beautiful recovery, and has recently expressed to me that she was free from pains and aches, and had better health than she had had for years.

Case 5th. Mrs. E., age 33, the mother of several children, sent for me in June, 1881, just a few mornings after I was sent for to see the case previously reported; found her with a hemorrhage from the uterus, which was soon under control by ice in the vagina and ergot by the mouth; a digital examination informed me at once of the cause of the trouble, for I found a dilatable os, and detected a foreign body in the cavity of the uterus. Mrs. E. informed me that she had been regular until two months previous, then missed her period once; two weeks from this time, while walking up town, her sickness came on her, (this was the version given me, but I have almost positive proof that it was a criminal abortion). The discharge still continuing quite profusely, she sent for her family physician, an intelligent gentleman and a warm personal friend of mine, who made her one visit, prescribed rest for her, and ordered ergot, gtts xx every three hours, believing it was only a continuation of her menstrual period, for the woman did not give the full history of the case as she afterwards did to me, the doctor being deceived as to what her actual condition was. Another week passed without any improvement in her condition, culminating in the hemorrhage for which I was called, in the absence of her family physician, who was then off on his summer vacation. After stopping the hemorrhage, as mentioned before, I tamponed the vagina, and on the following morning with my fingers and long pair of forceps, with the occasional use of the speculum, I removed several small pieces of putrid placenta, syringed out the vagina and uterus with a weak solution of carbolic acid, and gave her at once grs. x. of quinia and I gr. of opium ; from this day had no further trouble, and only made three subsequent visits. I have seen her on several occasions since, and she reports herself as having good health.

From the report of the cases under consideration (my line of treatment having been often mentioned) I make it an axiom : *in retained placenta, with hemorrhage or not, remove the cause and treat the symptoms as they arise.*

From my own experience with these and other cases coming under my notice, I have been impressed with the following facts:

1st. Their frequency.

2d. That a hemorrhage with a dilated and dilatable os, as a rule, means a foreign body in the cavity of the uterus.

3d. That in opium and quinia we have almost a specific (if given in proper doses) in controlling inflammation of the uterus and its appendages, setting up after operative interference with the same.

4th. That in the fingers we have a valuable instrument in removing the foreign bodies from the cavity of the uterus.

5th. The wonderful recuperative powers of women after the loss of an immense amount of blood.

6th. As long as we have a dilated os medicated injections into the cavity of the uterus are harmless, and in them we have a valuable aid in the treatment of the class of cases under consideration, especially subsequent to the removal of the retained placenta.

In conclusion, I would say that if I have succeeded in impressing on the minds of the members of this society the necessity of watching their patients after abortion, especially if there is a suspicion of retained placenta, this suspicion being verified or not before discharging them, either by digital or other forms of examination, doing this not only for their patients' sake, but also for their own reputation; I say sir, if I have succeeded in doing this, one of the main objects of my paper has been accomplished.

